# Stranding survey as a framework to investigate rare cetacean records of the north and north-eastern Brazilian coasts

**DOI:** 10.3897/zookeys.688.12636

**Published:** 2017-08-09

**Authors:** Alexandra Fernandes Costa, Salvatore Siciliano, Renata Emin-Lima, Bruna Maria Lima Martins, Maura Elisabeth Moraes Sousa, Tommaso Giarrizzo, José de Sousa e Silva Júnior

**Affiliations:** 1 Grupo de Estudos de Mamíferos Aquáticos da Amazônia (GEMAM). Programa de Capacitação Institucional, Setor de Mastozoologia, Coordenação de Zoologia, Museu Paraense Emílio Goeldi. Av. Perimetral, 1901, Terra Firme, 66077-530 Belém, PA, Brazil; 2 PPG em Ecologia Aquática e Pesca, Universidade Federal do Pará-UFPA, Instituto de Ciências Biológicas, Cidade Universitária José da Silveira Netto, Av. Augusto Corrêa n° 1, Guamá, 66075-110, Belém, PA, Brazil; 3 Instituto Oswaldo Cruz/FIOCRUZ, Pavilhão Mourisco – sala 122, Av. Brasil, 4365 - Manguinhos, 21040-360, Rio de Janeiro, RJ, Brazil; 4 PPG em Ecologia e Conservação da Biodiversidade, Universidade Estadual de Santa Cruz – UESC. Rodovia Jorge Amado, km 16-Pav. Maz de Menezes, 1º andar, sala 1DA, Salobrinho, 45662-900 Ilhéus, BA, Brazil; 5 PPG em Biologia Ambiental, Universidade Federal do Pará – UFPA, Campus de Bragança. Alameda Leandro Ribeiro s/n, Aldeia, 68600-000 Bragança, PA, Brazil; 6 Laboratório de Biologia Pesqueira e Manejo dos Recursos Aquáticos, Universidade Federal do Pará – UFPA, Av. Perimetral, 2651 Terra Firme, 66077-830 Belém, PA, Brazil

**Keywords:** Amazon, Balaenopteridae, beach survey, cluster analysis, Delphinidae, marine mammals distribution, Physeteridae, South America

## Abstract

Marine mammal stranding events are used as an important tool for understanding cetacean biology worldwide. Nonetheless, there are vast gaps of knowledge to be filled in for a wide range of species. Reputable information is required regarding species from large baleen whales to sperm and beaked whales, as well as pelagic dolphins. This paper describes new cetacean records from north and north-eastern Brazil, which are both the least surveyed areas regarding aquatic mammals. Regular beach surveys were conducted to recover cetacean carcasses along the coast of Pará beginning November 2005. At the coasts of the Maranhão and Piauí states, the surveys were conducted between 2003 and 2013. From 2003 to 2014, 34 strandings of cetaceans were registered. The study provides four additional species records’ in the area based on strandings (*Balaenoptera borealis*, *Balaenoptera physalus*, *Peponocephala electra*, and *Pseudorca crassidens*). A mass stranding of Guiana dolphins (*Sotalia guianensis*, N = 12), the most common species for the region, was reported for the first time. The records presented herein are of special concern, since they expand the knowledge on cetaceans from the Brazilian coast. In addition, this study conducted an analysis to verify the similarity between cetacean compositions described for north and north-eastern Brazil and the southern Caribbean region. The results showed a high similarity between these regions, proving the connection with the Caribbean cetacean fauna.

## Introduction

The Amazon Coastal Zone (ACZ), between 4°N and 4°S, presents a singular environment, characterized by the immense discharge of the Amazon River. ACZ is an unique aquatic ecosystem where the largest continuum of mangrove belt of the world is found (Souza-Filho et al. 2005). These characteristics combined with the recent coral reef ecosystem discovered in the mouth of the Amazon (Moura et al. 2016) make this region a top priority for conservation among Brazilian coastal environments. In this vast coastal area cetacean records have been documented only in the last decade. The north and north-eastern Brazil have been one of the least surveyed areas regarding aquatic mammals. Only one report made a compilation of cetacean records from this area (Siciliano et al. 2008) bringing the number of recorded species to 22, including large whales and dolphins. This study is an effort to increase this information after abovementioned study. Stranding events is one of the best ways to access data on biology and ecology of marine mammals (Norman et al. 2004, Evans et al. 2005, Pyenson 2010, Santos et al. 2010, Peltier et al. 2012, Covelo et al. 2015).

The cetacean fauna of Brazil has been subject to an extensive number of groups dedicated to conservation and research issues since the 1980’s. Because of this, a considerable amount of new information on cetacean distribution, biology and ecology has arisen (e.g. Pinedo et al. 2001; Zerbini et al. 2004). In 2005, with the establishment of the ‘Grupo de Estudos de Mamíferos Aquáticos da Amazônia (GEMAM)’ at the Museu Paraense Emílio Goeldi, the first systematic studies on aquatic mammals were initiated in the region.

Nevertheless, there are vast gaps of knowledge to be filled in for a wide range of species. From large baleen whales to sperm and beaked whales, as well as pelagic dolphins, reputable information is required. This is due, in part, to the lack of inventories over large areas of the Brazilian coast.

Studies on the occurrence of cetaceans around the world are made using different methodologies such as: strandings, historical records, opportunistic sightings, and dedicated surveys (Maldini et al. 2005, Lucena 2006, Toledo and Langguth 2009, Ramos et al. 2010, Authier et al. 2014, Prado et al. 2016). In other words, cetacean composition in a specific area could be assessed through strandings when other sources of information are not available (Maldini et al. 2005, Byrd et al. 2014, Jung et al. 2015). In this sense, Pyenson (2010) validated that cetacean death assemblages is a faithful method to document taxonomic richness and relative abundance of living cetaceans.

The main goals of this report are to: (1) present a variety of new cetacean records along the northern Brazilian coast, (2) increase baseline information on cetacean occurrence over 11 years (2003–2014); and (3) compare the cetacean composition in distinct areas along the north and north-eastern Brazilian coasts with southern Caribbean region, investigating the similarity between these sectors. The results of this study could open a promising new window for understanding the cetacean community structure in this vast stretch of coast.

## Materials and methods

### Study sites

The northern Brazilian coast has Cabo Orange as its limits to the north (05°N, 51°W), and São José Bay to the south (02°S, 44°15'W) and represent the Atlantic coastal sector. This vast coastal area extends for approximately 2250 km composed of different systems: mangrove forests, salt marshes, tidal sand flats, chenier sand ridges, coastal dunes, beach ridge barriers, and ebb tidal delta (Souza-Filho et al. 2008). The semi-diurnal macro-tidal regime with variations of approximately 4 m in Marajó Bay and 7.5 m in São José Bay (DHN, 2010) is another characteristic. Also, the ecosystem in this region is strongly influenced by rain season (high precipitation, low salinity, and high turbidity) and dry season (low precipitation, high salinity, and low turbidity) (Moraes et al. 2005). The freshwater input along this coastline is different. In the northern the influence of the enormous discharge of the Amazon River dominates, while in the eastern coast of Maranhão and in the coastline of Piauí, the principal effect comes from the discharge of Parnaíba River (Szczygielski et al. 2014).

**Figure 1.**
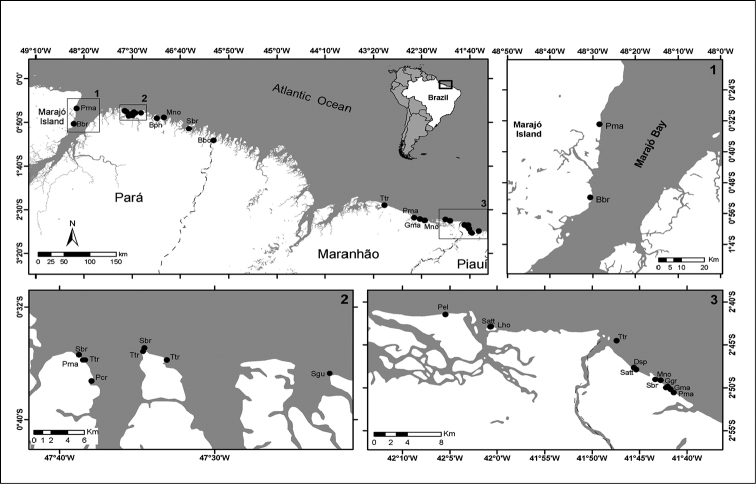
Study sites. Surveyed sectors on north and north-eastern Brazilian coast: **1** Marajó Bay **2** Eastern Pará state and **3** Maranhão and Piauí coastlines encompass part of Parnaíba Delta. Black dots representing stranding locations. *Physeter macrocephalus* (Pma), *Balaenoptera brydei* (Bbr), *Balaenoptera physalus* (Bph), *Megaptera novaeangliae* (Mno), *Steno bredanensis* (Sbr), *Balaenoptera borealis* (Bbo), *Tursiops truncatus* (Ttr), *Globicephala macrorhynchus* (Gma), *Pseudorca crassidens* (Pcr), *Sotalia guianensis* (Sgu), *Peponocephala electra* (Pel), *Stenella attenuata* (Sat), *Lagenodelphis hosei* (Lho), *Delphinus* sp. (Dsp) and *Grampus griseus* (Ggr).

The study site of this report is divided into three sampling sectors: (1) Marajó Bay (MB), (2) Eastern Pará state (EP) and (3) Maranhão/Piauí coastline (MA/PI), encompassing part of Parnaíba Delta (Fig. 1).

Marajó Bay is located on the eastern coast of Marajó Island (Fig. 1, frame 1). This bay is formed mainly by the discharges of Pará and Tocantins rivers and Guajará Bay and is influenced by oceanographic processes such as superficial saline intrusion during dry season under low river discharges.

In the eastern coast of Pará (Fig. 1, frame 2) and north-west coast of Maranhão the coastline is designated as Amazon macrotidal coastal zone. This region encompass almost 70% of Brazilian mangrove forests, possess low relief (0 to 80 m), broad coastal plain (with up to 70 km wide) and large continental shelf (200 km approximately). This coastline is extremely irregular and forms numerous bays and small estuaries, the area is influenced for a macrotidal regime (Souza-Filho 2005).

Maranhão and Piauí coastlines, encompassing the Parnaíba Delta (Fig. 1, frame 3), are considered a semiarid region. Parnaíba River discharge into the Atlantic Ocean forming a delta with five bays: Tutóia, Caju, Melancieiras, Canárias and Igaraçu (Moreira and Mavigner 2007). Parnaíba Delta comprises a mesotidal coast with beaches with up to 200 m wide and large areas of mangrove forests (Szczygielski et al. 2014).

Stranding events in these areas were regularly monitored between 2005 and 2014 in MB and EP sampling sectors, and from 2003 to 2013 in MA/PI sector (Fig. 1, see frame 1 and 2). Every two-week period, the vast stretch of coastline is reached by boat and four-wheel vehicle or, alternatively, monitored by foot. At least three field monitors take part in the field and on dedicated surveys the same routes were covered.

On occasion, the team was called to respond to live strandings or carcasses washed ashore. These events, as they are exclusive in their nature and circumstances, are validated with voucher samples, photographs, and other sources of original information.

The carcasses were identified in the field following specific features (e.g. colouration, shape of the head and fins/flippers, number of teeth). In such specific cases as *Delphinus* sp. and *Megaptera novaeangliae*, the identification was made posteriorly through cranial measures and scapular features, respectively.

Complete and incomplete carcasses were recovered and their skeletons and soft tissues, after preparation, are deposited at the Mammal Collection from Museu Paraense Emílio Goeldi (MPEG), located in Belém, Pará, Brazil. Table 1 lists the records of cetaceans stranded from 2003 to 2014 on the northern and north-eastern Brazilian coast according to the three sampling sectors, including voucher numbers from the abovementioned collection.

**Table 1.** Cetacean records from 2003 to 2014 on the northern Brazilian coast.

Species by Family	Category	TL (m)	Sex	Sector	Date	Voucher specimens
**Balaenopteridae**						
*Balaenoptera borealis*	LS	10.32	F	EP	13/09/2008	MPEG 39691
*Balaenoptera brydei*	CA	4.20	NI	MB	15/09/2012	MPEG 42154
*Balaenoptera physalus*	LS	14.90	M	EP	21/01/2010	MPEG 39690
*Megaptera novaeangliae*	CA	16.70	F	MA/PI	15/01/2003	-
	CA	-	NI	MA/PI	2005	-
	CA	10.00*	M	EP	08/10/2008	MPEG 39692
	CA	13.00*	NI	MA/PI	22/05/2009	MPEG 42184
**Physeteridae**						
*Physeter macrocephalus*	CA	11.80	F	MA/PI	02/02/2010	MPEG 42088
	CA	-	F	MA/PI	14/05/2010	MPEG 42173
	LS	10.50	F	EP	07/04/2014	MPEG 42177
	CA	4.22	NI	MB	08/08/2014	MPEG 42178
**Delphinidae**						
*Delphinus* sp.	CA	2.06	NI	MA/PI	12/08/2011	MPEG 42095
*Globicephala macrorhynchus*	CA	-	NI	MA/PI	2009	-
	CA	-	NI	MA/PI	15/01/2009	MPEG 42128
*Grampus griseus*	CA	-	NI	MA/PI	13/04/2011	MPEG 42130
*Lagenodelphis hosei*	LS	-	M	MA/PI	03/04/2009	MPEG 42080
*Peponocephala electra*	CA	-	NI	MA/PI	2007	MPEG 42067
	CA	-	NI	MA/PI	19/06/2008	MPEG 42069
*Pseudorca crassidens*	CA	3.30	NI	EP	20/04/2012	MPEG 42132
*Sotalia guianensis*	LS	-	NI	EP	31/01/2013	-
*Stenella attenuata*	CA	-	NI	MA/PI	14/03/2009	MPEG 42077
	CA	1.59	M	MA/PI	10/12/2009	MPEG 42085
*Steno bredanensis*	CA	-	NI	EP	19/04/2009	MPEG 39635
	CA	-	NI	MA/PI	15/08/2011	MPEG 42096
	CA	-	NI	EP	20/11/2011	MPEG 42066
	CA	-	NI	EP	20/11/2011	MPEG 42102
	CA	2.68	M	EP	04/04/2012	MPEG 42131
	CA	-	NI	MA/PI	23/08/2013	MPEG 42176
*Tursiops truncatus*	CA	-	NI	MA/PI	24/04/2009	MPEG 42129
	CA	-	NI	MA/PI	26/07/2009	MPEG 42081
	CA	-	NI	MA/PI	05/03/2010	-
	CA	-	NI	EP	12/03/2010	MPEG 39612
	CA	3.17	M	EP	13/02/2013	MPEG 42174
	CA	2.87	F	EP	04/03/2013	MPEG 42175

Species were divided by family, details of specimens and stranding. Abbreviations: TL= total length in meters; Sex= female, male and not identified (NI); Category of stranding - live (LS) and carcass (CA), Date, sector and location of events; MB= Marajó bay; EP= Eastern Pará state; MA/PI= Maranhão/Piauí coastline * represents the estimated TL (specimens missing the skull)

### Data source and analysis

The similarity between the cetacean composition described in this study and the most representative surveys conducted in the north (Siciliano et al. 2008) and part of the north-eastern Brazilian coasts (Alves-Júnior et al. 1996, Motta et al. 2008, Meirelles et al. 2009), in addition to the Caribbean region (Romero and Creswell 2005, Luksenburg 2014a, 2014b), was analyzed.

Similarities between the stranding and sighting records of cetaceans reported in seven articles and the present study was accomplished through an analysis of similarity by the Jaccard index based on the presence or absence of species. A similarity profile (SIMPROF) test was performed to detect the significantly different groups using the default of 1.000 permutations for the mean similarity profile and 999 permutations for the simulated profile, with a significance level of 0.05. Multivariate analyses were performed using PRIMER 6.0 (PRIMER-E Ltd., Plymouth, U.K) (Clarke and Gorley 2006).

## Results

### Species richness and diversity

The present study recorded rare strandings of some cetacean species (e.g. mass stranding of *Sotalia guianensis*), and the first occurrence of these cetaceans in northern coast: 1. Sei whale, *Balaenoptera borealis* Lesson, 1828, 2. Fin whale, *Balaenoptera physalus* (Linnaeus, 1958), 3. Melon-headed whale, *Peponocephala electra* (Gray, 1846) and 4. False killer whale, *Pseudorca crassidens* (Owen, 1846).

Species richness was represented for 15 taxa, within three distinct families: Balaenopteridae (*B. borealis*, *B. physalus*, *B. brydei*, *M. novaeangliae*), Physeteridae (*P. macrocephalus*) and Delphinidae (*Delphinus* sp., *G. macrorhynchus*, *G. griseus*, *Lagenodelphis hosei*, *P. electra*, *P. crassidens*, *S. guianensis*, *S. attenuata*, *S. bredanensis*, *T. truncatus*) (Table 1).

### Spatial distribution

In these sampling sectors (MB = Marajó bay; EP = Eastern Pará; MA/PI = Maranhão/Piauí coastline) stranding events were distributed unequally. The spatial distribution among sectors showed that the majority of strandings occurred at the Maranhão/Piauí coastline 54.5% (N = 18), on the north-eastern coast of Brazil. The EP occupies the second place, with 39.4% (N = 13) and MB with only 6.1% (N = 2) of rare cetacean records.

Differences in strandings by family and sector were analyzed in order to evaluate patterns along the distinct sectors of this coastline. Members of the Delphinidae family were the most representative in the frequency of strandings, comprising 66.7% (N = 22), followed by Balaenopteridae 21.2% (N = 7) and Physeteridae 12.1% (N = 4).

### Cetacean Composition

#### Balaenopteridae

##### *Balaenoptera borealis* Lesson, 1828

**Sei whale**

**Figure 2.**
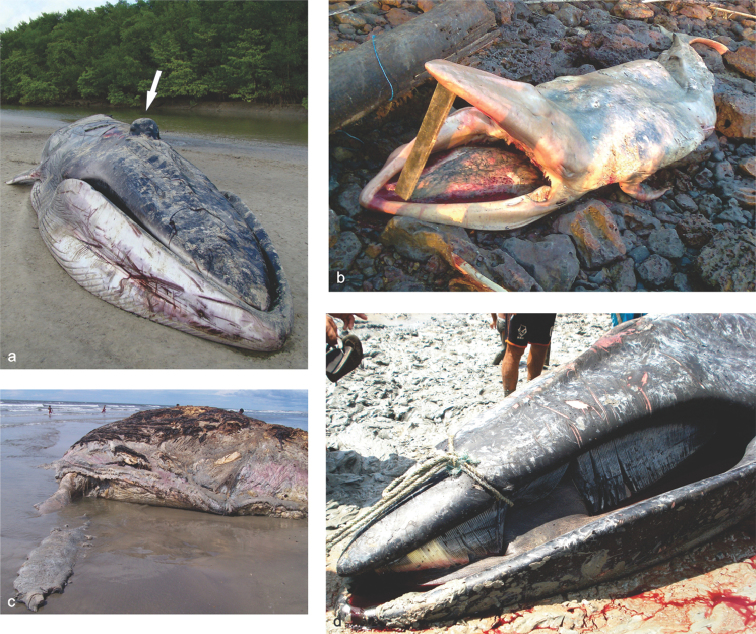
Stranding events: large baleen whales. **a** Fin whale *Balaenoptera physalus* stranded at Baía do Japerica, São João de Pirabas municipality (0°45'S, 47°4.2'W), eastern coast of the Pará state. Note the good condition of the carcass and the strange swelling on the top of the head (see white arrow) (photo by GEMAM/MPEG) **b** A newborn Bryde’s whale *Balaenoptera brydei* stranded on the east coast of Marajó island (0°51.6'S, 48°30'W), Pará on 15 September 2012 (photo by GEMAM/MPEG) **c** Humpback whale *Megaptera novaeangliae* stranded at Praia do Rio Novo, Tutóia municipality, Maranhão state (2°42.6'S, 42°26.4'W), note the absence of skull on the carcass (photo by A.F. Costa) **d** Sei whale *Balaenoptera borealis* stranded at Fernandes Belo, Viseu municipality (1°10.8'S, 46°5.4'W), the particular coloration of the baleen plates and slightly arched head are diagnostic characters to identify the species (photo by DEMA/PA).

On 13 September 2008 a large whale stranded alive on the eastern Pará state (EP). The specimen (MPEG 39691) had features characteristically attributed to Sei whales: a dark body with patches of lighter gray, both sides of head evenly dark, head slightly arched, throat grooves ending just behind the flippers, grey-black baleen plates with a metallic sheen, lighter plates near the front of the mouth and a sickle-shaped dorsal fin. After unsuccessful trials of pushing the whale back to the sea, it died (Fig. 2d). The necropsy revealed a large amount of mud in the stomach, probably originated from the mangrove sediments nearby the stranding site.

##### *Balaenoptera brydei* Olsen, 1913

**Bryde’s whale**

On 15 September 2012 a decomposed carcass of a newborn *B. brydei* washed ashore on the rocky shore of Praia de Água Boa, Salvaterra, Marajó bay (MB), Pará (Fig. 2b). Partial skeleton and tissue samples were collected (MPEG 42154).

##### *Balaenoptera physalus* (Linnaeus, 1958)

**Fin whale**

On January 2010, a male Fin whale *B. physalus* was reported stranded on the eastern Pará state (EP) in Baía do Japerica, São João de Pirabas municipality. Fishermen and residents sighted the live specimen entering the estuary but a few days later the carcass was found stranded in a different location. The fresh specimen had a large callosity on the top of the head (Fig. 2a). The nearly complete skeleton, including skull and mandibles, is deposited in the mammal collection (MPEG 39690).

#### *Megaptera novaeangliae* (Borowski, 1781)

**Humpback whale**

The first evidence of a Humpback whale *M. novaeangliae* in Piauí is represented by a female stranded at Praia da Pedra do Sal, Parnaíba on 15 January 2003. Subsequently, in 2005, locals collected a scapula at Praia da Carnaubinha, Luís Correia municipality. On May 2009, a decomposed carcass missing the skull was found stranded in the Praia do Rio Novo, Tutóia municipality, voucher No MPEG 42184 (Fig. 2c).

#### Physeteridae

##### *Physeter macrocephalus* Linnaeus, 1758

**Sperm whale**

**Figure 3.**
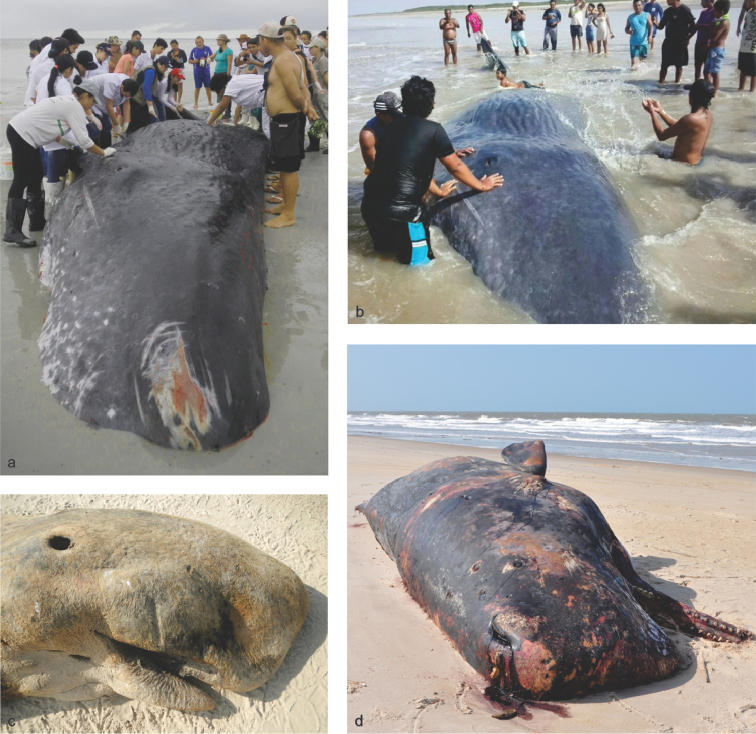
Stranding events: sperm whales. **a** Sperm whale *Physeter macrocephalus* (MPEG 42166) stranded at Praia do Crispim, Marapanim municipality (0°34.8'S, 47°38.4'W) eastern coast of Pará state few hours after death (photo by A.F. Costa) **b** The same specimen (MPEG 42166) still alive with local people trying to help (internet file) **c** A newborn Sperm whale (MPEG 42178) stranded at the east coast of Marajó island, Praia do Camburupy (0°32.4'S, 48°28.2'W) on 2014, note the absence of teeth characteristic of a very young specimen (photo by A.F. Costa) **d** A female Sperm whale (MPEG 42088) stranded at Praia da Pedra do Sal, Parnaíba municipality, Piauí state coastline (2°49.8'S, 41°41.4'W) on 2010 (photo by A.F. Costa).

On 02 February 2010 a female with TL = 11.80 m (MPEG 42088) stranded at Praia da Pedra do Sal, Parnaíba municipality, Piauí state (Fig. 3d). The carcass was moderately fresh, had numerous bites of cookie-cutter shark (*Isistius* sp.) over the body and evidence of endoparasites in the blubber. The remains of a second specimen, also a female, stranded at Praia do Baixo da Boia, Paulino Neves municipality, Maranhão state on 14 May 2010. Some vertebrae and chevrons with deformations were recovered (MPEG 42173). On 07 April 2014, a live-stranded Sperm whale was recorded for the first time along the Pará coastline. The specimen was a female with TL = 10.50 m (MPEG 42166) that came ashore at Praia do Crispim (Fig. 3a, b), had a large amount of squid beaks in its stomach and intestines. On 08 August 2014, a very young specimen (TL = 4.22 m) stranded at east coast of the Marajó Island in Praia do Camburupy, Soure municipality. Sex was not determined due to the advanced decomposition of the carcass (Fig. 3c).

#### *Delphinidae*

##### Oceanic species

###### *Delphinus* sp. Gray, 1828

**Common dolphin**

On 12 August 2011 a decomposed delphinid carcass was found at Praia da Pedra do Sal, Parnaíba municipality, Piauí (MA/PI coastline) (MPEG 42095). This is the very first record of a Common dolphin for this portion of the Brazilian coast.

###### *Lagenodelphis hosei* Fraser, 1956

**Fraser’s dolphin**

**Figure 4.**
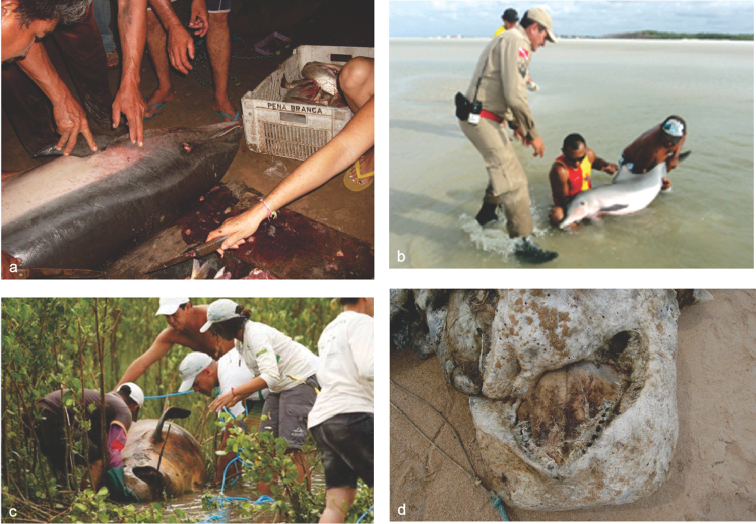
Stranding events: small and medium-sized cetaceans. **a** The carcass of a Fraser’s dolphin, *Lagenodelphis hosei* was cut down on the village of Carnaubeira, Araioses municipality (2°42.6'S, 42°0.6'W) and consumed locally (photo PROCEMA files) **b** A group of 12 live Guiana dolphins *Sotalia guianensis* were found trapped (photo showed just one specimen) in a tidal channel alongside beach, Salinópolis municipality, eastern Pará (0°36'S, 47°22.2'W) **c** A False killer whale *Pseudorca crassidens* stranded on the north-eastern coast of Pará, Praia de Marudá, Marapanim municipality (photo GEMAM files) **d** A Short-finned pilot whale *Globicephala macrorhynchus* stranded in advanced stage of decomposition at Praia da Pedra do Sal, Parnaíba municipality (2°40.8'S, 42°31.2'W), the shape of the head and number of teeth were diagnostic to identify the species (photo PROCEMA files).

On the coast of the Maranhão state, at Praia da Barrinha, Canárias Island, the carcass of a Fraser’s dolphin *Lagenodelphis hosei* was found fresh by fishermen on 03 April 2009, after it presumably live-stranded on the beach. The carcass was divided up at the village of Carnaubeira, Araioses municipality (Fig. 4a) and consumed locally (meat slices were found drying on clothes-line in villagers’ houses).

###### *Stenella attenuata* (Gray, 1846)

**Pantropical spotted dolphin**

The Pantropical spotted dolphin *S. attenuata* is represented by two records from the MA/PI sector, both from 2009. Bone remains were found at the Canárias Island, Praia da Barrinha, Maranhão (MPEG 42077) and a decomposed carcass of a male was found at Praia da Pedra do Sal, Parnaíba municipality, Piauí (MPEG 42085), with marks of human interaction.

##### Blackfish

A Short-finned pilot whale *Globicephala macrorhynchus* Gray, 1846 was found stranded in an advanced state of decomposition in the Parnaíba municipality, Piauí (MA/PI), on 15 January 2009 (MPEG 42128). Identification was made possible through a combination of head features and tooth counts (Fig. 4d). In August 2009, during beach surveys at the Maranhão coast, the photographic record of a mounted skeleton on display in a beach tent was confirmed as *G. macrorhynchus* at the locality of Paulino Neves (MA/PI).

An incomplete Risso’s dolphin skull *Grampus griseus* (G. Cuvier, 1812) (MPEG 42130) found buried was recovered at Praia da Pedra do Sal, Parnaíba municipality, Piauí, MA/PI sector, on 13 April 2011.

Two stranding records of Melon-headed whale *Peponocephala electra* (Gray, 1846) were reported for the Ilha do Caju and Delta do Parnaíba (MA/PI). Only bone remains were recovered (MPEG 42067, MPEG 42069).

The first record of a False killer whale *Pseudorca crassidens* (Owen, 1846) on the Pará coastline occurred on 20 April 2012. The carcass was found in the mudflats at Praia de Marudá (Fig. 4c), Marapanim municipality, eastern Pará (EP). The specimen (MPEG 42132) had marks of human interaction; the peduncle was cut-off and most of the teeth were removed.

#### Coastal species

##### *Sotalia guianensis* (Van Bénedén, 1864)

**Guiana dolphin**

On 31 January 2013, 12 Guiana dolphins *S. guianensis* were reported as trapped in a tidal channel alongside Praia da , Salinópolis municipality, eastern Pará state (Fig. 4b, see Suppl. materials 1 and 2). They were all rescued by life savers and locals, who carried the dolphins along 700 m to the receding waters during the low tide. This event could be considered a mass stranding of Guiana dolphins, so far the first reported for the northern coast of Brazil, and presumably in the country.

##### *Steno bredanensis* (G. Cuvier in Lesson, 1828)

**Rough-toothed dolphin**

On 04 April 2012, the carcass of a mature male (MPEG 42131) Rough-toothed dolphin *S. bredanensis* stranded in a moderate state of decomposition showed bites and a huge scar on the caudal peduncle, which could indicate interaction with long line fisheries. New records of rough-toothed dolphins were confirmed from EP, MPEG 39635 (Praia da Princesa, Algodoal Island, Maracanã municipality), MPEG 42066/ MPEG 42102 (Praia do Crispim, Marapanim municipality) and from MA/PI coastline, MPEG 42096/MPEG 42176 (Praia da Pedra do Sal, Parnaíba municipality).

##### *Tursiops truncatus* (Montagu, 1821)

**Bottlenose dolphin**

Five new records of Bottlenose dolphin *T. truncatus* were reported after the previous review by Siciliano et al. (2008) (see Table 1 for complete information). On 5 March 2010, in Praia da Travosa, Santo Amaro municipality, Maranhão (MA/PI) (See Suppl. material 3), a young Bottlenose dolphin *T. truncatus* was found live-stranded on a beach and was rescued by bathers, locals and fishermen.

### Cluster analysis

**Figure 5.**
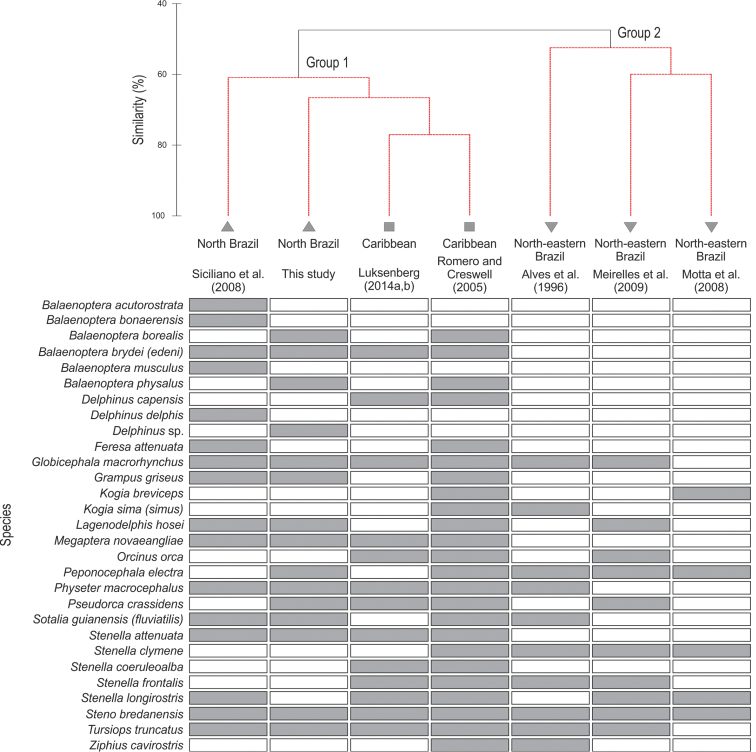
Cluster analysis of cetacean stranding and sighting records. Diagram of cetaceans reported from the north-eastern (inverted triangle) and north coast of Brazil (triangle) and southern Caribbean (square), reported in seven articles and the present study. The two distinct groups formed (Group 1 and 2) are indicated. A similarity profile (SIMPROF) permutation test highlights dashed clusters that show significant internal structure. Shaded cells indicate occurrence of species.

The cluster analysis indicated that the cetacean composition from the Caribbean and north Brazilian coast form a single group (Group 1) that differs significantly from the group formed at north-eastern Brazil (Group 2) (Fig. 5).

The richness of species varied from 13 to 24, with 29 species in totality. Group 2 was defined by adding stranding information from north-eastern Brazil (Alves-Júnior et al. 1996, Motta et al. 2008, Meirelles et al. 2009). Species richness ranged from 5 to 10, with 15 species in total.

## Discussion

Stranding data are an important source to understanding the biology and ecology of marine mammals (Geraci and Lounsbury 1993, Moura and Siciliano 2012, Covelo et al. 2015). Indeed, the records presented herein are of particular importance, as they clarify the composition and richness of the cetacean community in a vast stretch of the Brazilian coast. Current data, with the addition of four species to the previous list (Siciliano et al. 2008), expanded this number to 26 aquatic mammal species. This study is in accordance to (Pyenson 2010) which suggests that stranding records at extensive latitude gradients (>1000 km) and for long time periods, provide an useful and faithfully method to understand richness and cetacean community in a particular area than the sighting surveys (e.g. line-transect method). Maldini et al. (2005) analyzing the composition of cetaceans of the Hawaiian Islands suggested that the stranding data reflects the species composition found in living animals surveys and defends the usefulness use of stranding data as an important source of information in the absence of other data. Marine mammalogists assumed that stranding events could be affected by oceanographic features such as bottom topography, tides, currents, winds and seasonal patterns (Norman et al. 2004). Carcasses can be moved by currents and winds along the coast and the oceanographic differences observed in this vast area of the north and north-eastern Brazilian coast should be considered important factors on stranding distributions. This coastline is singular and considered a continuous belt of mangroves with approximately 7591km^2^ (Souza-Filho 2005) which complicates access and, consequently the rescue and reports of stranded marine mammals. The macrotidal characteristic observed in the Marajó Bay and Eastern Pará sectors is another factor that should explain the low frequency of strandings especially of small and medium-sized cetaceans. The dynamic of macrotidal regime may in a few hours carry the carcass of a small cetacean without it could be seen in these vast uninhabited areas.

**Table 2.** Localities of marine mammal strandings and other localities mentioned in this study.

Locality	Municipality	Lat / Long	Description (species, others)
Cabo Orange	Oiapoque	5°N, 51°W	northern Brazilian coast to the west
Ponta do Tubarão	Baía de São José	4°00'S, 43°00'W	northern Brazilian coast to the east
Fernandes Belo	Viseu	1°10.8'S, 46°5.4'W	*Balaenoptera borealis*
Praia de Água Boa	Salvaterra	0°51.6'S, 48°30'W	*Balaenoptera brydei*
Baía do Japerica	São João de Pirabas	0°45'S, 47°4.2'W	*Balaenoptera physalus*
Praia da Pedra do Sal	Parnaíba	2°49.15'S, 41°42.8'W	*Megaptera novaeangliae*
Praia da Carnaubinha	Luís Correia	2°54.77'S, 41°30.66'W	*Megaptera novaeangliae*
Praia do Rio Novo	Tutóia	2°42.6'S, 42°26.4'W	*Megaptera novaeangliae*
Praia de Fora	Quatipurú	0°44.4'S, 46°57'W	*Megaptera novaeangliae*
Praia da Pedra do Sal	Parnaíba	2°49.8'S, 41°41.4'W	*Physeter macrocephalus*
Praia do Baixo da Boia	Paulino Neves	2°37.2'S, 42°38.4'W	*Physeter macrocephalus*
Praia do Crispim	Marapanim	0°34.8'S, 47°38.4'W	*Physeter macrocephalus*
Praia do Camburupy	Soure	0°32.4'S, 48°28.2'W	*Physeter macrocephalus*
Praia da Pedra do Sal	Parnaíba	2°47.4'S, 41°45'W	*Delphinus* sp.
Praia da Barrinha	Araioses	2°42.6'S, 42°0.6'W	*Lagenodelphis hosei*
Praia da Barrinha	Araioses	2°42.6'S, 42°0.6'W	*Stenella attenuata*
Praia da Pedra do Sal	Parnaíba	2°47.4'S, 41°45'W	*Stenella attenuata*
Praia da Pedra do Sal	Parnaíba	2°49.8'S, 41°41.4'W	*Globicephala macrorhynchus*
Paulino Neves	Paulino Neves	2°40.8'S, 42°31.2'W	*Globicephala macrorhynchus*
Praia da Pedra do Sal	Parnaíba	2°49.8'S, 41°42'W	*Grampus griseus*
Delta do Parnaíba	-	-	*Peponocephala electra*
Ilha do Caju	Araioses	2°41.4'S, 42°5.4'W	*Peponocephala electra*
Praia de Marudá	Marapanim	0°37.2'S, 47°37.8'W	*Pseudorca crassidens*
Praia da	Salinopólis	0°36'S, 47°22.2'W	*Sotalia guianensis*
Praia de Ajuruteua	Bragança	0°49.2'S, 46°36'W	*Steno bredanensis*
Praia da Princesa	Maracanã	0°34.2'S, 47°34.8'W	*Steno bredanensis*
Praia do Crispim	Marapanim	0°34.8'S, 47°38.4'W	*Steno bredanensis*
Praia do Crispim	Marapanim	0°34.8'S, 47°38.4'W	*Steno bredanensis*
Praia da Pedra do Sal	Parnaíba	2°49.2'S, 41°42.6'W	*Steno bredanensis*
Praia da Pedra do Sal	Parnaíba	2°48'S, 41°43.8'W	*Steno bredanensis*
Praia da Princesa	Maracanã	0°34.2'S, 47°34.8'W	*Tursiops truncatus*
Praia da Moita Verde	Araioses	2°43.8'S, 41°48'W	*Tursiops truncatus*
Furo Velho	Maracanã	0°35.4'S, 47°33'W	*Tursiops truncatus*
Porto do Sossego	Marapanim	0°36.6'S, 47°37.8'W	*Tursiops truncatus*
Praia da Travosa	Santo Amaro	2°21'S, 43°15'W	*Tursiops truncatus*
Baía do Capim	-	1°76'S, 44°83'W	Sperm whale stranding at Maranhão coast

The spatial distribution of species in the three sectors (MB, EP and MA/PI coastline) provides relevant information on their occurrence through stranding notifications. Small pelagic and neritic-pelagic cetaceans have been recorded in higher numbers along the EP and MA/PI sectors. In contrast, the MB area and adjacent coastline are influenced by the Amazon and Pará/Tocantins Rivers discharges, thus presumably limiting the occurrence and subsequent stranding of pelagic species. It should be considered that areas of difficult access and sparsely populated in the Amazon coastal zone, as the eastern Pará and the western Maranhão states, could represent potential circumstances for the low stranding notifications in these sectors. Although the systematic monitoring effort was lower at Maranhão/Piauí coastline, this area resulted to be singular due to the exclusive cetacean records of pelagic and neritic-pelagic species (e.g. *Lagenodelphis hosei*, *Peponocephala electra*). Despite the surveys were more dedicated in the MB sector, the frequency of uncommon strandings (e.g. oceanic species) were lower. However, single strandings of *Sotalia guianensis*, *Inia geoffrensis* and *I. araguaiaensis* in MB are not included in this report as they are commonplace and will be analyzed separately.

Earlier records of balaenopterids on the Amazon coastal zone were performed by Siciliano et al. (2008) and Silva et al. (2013). Previous revised information on stranding and sighting records of baleen whales along the Brazilian coast post-1997 (Siciliano et al. 2011) emphasizes the relevance of the current records. They are the very first evidence of the Fin and Sei whales occurrence along the northern Brazilian coast. The stranding intervals in each case could be informative of their movements and migration periods. The Sei whale was stranded in September 2008, during the peak migration period of southward migration (August–October) (sensu Best and Lockyer 2002). In accordance to these authors, the Viseu specimen (MPEG 39691), TL = 10.32 m in length, is acceptable as the size of immature females caught off South Africa (range 10.7–13.7 m). The Fin whale stranding demands a more complex interpretation, since the specimen became stranded in January, by the time most large whales have already reached Antarctic and sub-Antarctic waters. Siciliano et al. (2011) reported on a Fin whale sighted in January 2004 off the Santos Basin. Our present record poses an intriguing question, whether this whale was of a southern hemisphere origin or truly representative of a northern hemisphere stock.

The newborn Bryde’s whale recorded in Praia da Água Boa (MB) on 15 September 2012 is a remarkable record, since very limited information exists on the pregnancy and time of birth of this tropical balaenopterid and, in Brazil, information on the calving season is almost non-existent (Santos et al. 2010). Moura and Siciliano (2012) report on two newborns stranded only a few months apart in south-eastern Brazil (August and September). Our record was in September, agreeing with these authors’ previous observations and, thus, suggesting the same period of the year as a calving season for Bryde’s whales off the northern Brazilian coast. Records of Humpback whales herein expands significantly the presence of the species westwards the north-eastern Brazilian coast. Pretto et al. (2009) reported the first occurrence of *M. novaeangliae* for eastern coast of Pará at Praia de Fora, Quatipuru municipality.

In relation to odontocetes, four additional Sperm whales records are included from the Pará state and Maranhão/Piauí coastlines. One of them is of a very young specimen (TL = 4.22 m) stranded at east coast of the Marajó Island. This record strongly suggests the existence of calving off in the northern Brazilian coast. Sperm whales are a regular component of cetaceans found stranded on the north-eastern coast of Brazil. For example, Meirelles et al. (2009) provided 26 records for the Ceará state alone over the period from 1993 to 2005. The sample includes specimens of all sizes, from a 3.53 m newborn to a 18.10 m bull male. It was assumed that the strandings took part mostly in spring, summer, and winter months, influenced by strong north-western winds, when carcasses have more chance of coming ashore. Magalhães et al. (2008) have contributed with two records of Sperm whales for the Maranhão coastline, during their short-term surveys from 2004 to 2006. Other records for the north-eastern coast include a newborn stranded near of Baía do Capim (1°76'S, 44°83'W), Maranhão coast (S3 File), and four other specimens reported by Tosi et al. (2006). In a broader perspective, it is highly recommended to conduct future surveys in order to estimate Sperm whale density and abundance off the north and north-eastern Brazilian coasts. The structure of this Sperm whale population in relation to more southerly aggregations in Brazil deserves further studies and agreed with literature which report that females and immature males inhabit lower latitudes in subtropical and tropical oceans (Whitehead 2009).

Blackfish records were represented by the False killer whale, Melon-headed whale, Short-finned pilot whale, and Risso’s dolphin recovered in distinct areas. These specimens improve the knowledge on blackfish occurrence off the northern Brazilian coast. The False killer listed in this study is noteworthy; this is the first record of this species in the region. The specimen (MPEG 42132) had marks of human interaction; the peduncle was cut-off and most of the teeth were removed. This strongly suggests interactions with the pelagic fishery activity. Such marks were also observed in a rough-toothed dolphin stranded in eastern Pará (MPEG 42131) and on a pantropical spotted dolphin found on Piauí (MPEG 42085), suggesting that this kind of negative interaction may be more common than previously thought. Luksenburg (2014a) has informed on injuries found in live small cetaceans off Aruba, in the southern Caribbean. The author reported that of the 18 False killer whales individuals presenting injuries, six (33.3%) showed wounds that were probably caused by human activities. Two of these injuries were most likely caused by fishing gear and two others were most likely the result of a propeller hit. Further investigation on the impact of such fishing operations off northern and north-eastern Brazil on False killer whales and other smaller pelagic delphinids is highly needed.

The records of Bottlenose, Rough-toothed, and Common dolphins indicate their regular presence in neritic waters off northern Brazil, where then are referred by the fishermen as ‘tuninas’. The Common dolphin specimen recorded at Praia da Pedra do Sal is certainly of special interest as so little is known of this population inhabiting the north-eastern coast (see Tavares et al. 2010 for more details).

The evidence of consumption of a fresh Fraser’s dolphin denotes further investigation on the use of marine bushmeat along small fishing communities of north-eastern Brazil. Tosi et al. (2008) have reported a similar episode involving Fraser’s dolphin consumption in the coast of Maranhão. Even if these represent fortuity events, dolphin and whale meat consumption should be more deeply investigated in such villages in future surveys.

Regarding coastal species, a mass stranding of Guiana dolphins was reported for the first time, based on an incident recorded in Salinópolis (EP), on January 2013. The coastline of Pará possesses certain unique features, such as very strong tides that cause accelerated movement of the sand banks and tidal channels. It seems plausible to believe in a natural condition characterized by this muddy beach and fast lowering tide acting as a trap. Although Guiana dolphins are familiar with flat waters and tide dynamism, the particular location of the Praia da could have acted as a natural trap. Nonetheless, the singularity of this event is worth mentioning and adds a new feature to the natural behavior of Guiana dolphins.

The cetacean fauna off the north and north-eastern Brazilian coast is much richer and diverse that previously thought, including both residents of tropical oligotrophic waters and highly-migrating baleen whales. Species associated to productive environments are also included, such as Bryde’s whales and the Common dolphin. As pointed out by the present survey, the connection with the southern Caribbean cetacean fauna seems plausible and deserves further investigation. This is particularly relevant for understanding the affinity of north Brazilian Bryde’s whale and Common dolphin populations with the adjacent Venezuela regional aggregations of these species (Galindo 2007). There is a lack of information on countries of Wider Caribbean Region as Guyana, Suriname, and French Guiana, however, surveys developed at Guiana’s coastal province supply important records on cetacean composition (Manocci et al 2013, Boer 2015). Such a relationship is highly expected and could potentially connect the north Brazilian cetacean fauna with that from the southern Caribbean.

## Conclusions

Strandings of uncommon cetacean species and a live beached Sperm whale are recorded for the first time along the north coast of Brazil. Balaenopterids records (*Balaenoptera borealis* and *B. physalus*) presented herein are of particular importance and include the very first of its category on the northern coast of Brazil providing new insights on migration movements on the southern hemisphere.

Evidence of anthropogenic injuries inflicted to cetaceans and also the consumption of marine bushmeat is reported to the north-eastern coast of Brazil. Although the proximity of the northern and the north-eastern Brazilian coasts, results showed that cetacean fauna in northern is more similar with the Caribbean region. Further investigation on movements and distribution patterns should be encouraged.

In this context, beach surveys and stranding network definitely provide relevant information on marine mammals’ richness and diversity is this vast region. The cetacean fauna of north and north-eastern Brazil combines different elements from tropical marine ecosystems under the influence of the most powerful river of the world. Such dynamism requires a proportional research effort to better evaluate and understand its own complexity.

## Supplementary materials

Supplementary material 1 S1 Movie Data type: MOV file Explanation note: Dolphins trapped at Praia da , Salinópolis (MOV). This dataset is made available under the Open Database License (http://opendatacommons.org/licenses/odbl/1.0/). The Open Database License (ODbL) is a license agreement intended to allow users to freely share, modify, and use this Dataset while maintaining this same freedom for others, provided that the original source and author(s) are credited.Alexandra Fernandes Costa, Salvatore Siciliano, Renata Emin-Lima, Bruna Maria Lima Martins, Maura Elisabeth Moraes Sousa, Tommaso Giarrizzo, José de Sousa e Silva Júnior

Supplementary material 2 S2 Movie Data type: MOV file Explanation note: Rescue Guiana dolphins trapped at Praia da , Salinópolis (MOV). This dataset is made available under the Open Database License (http://opendatacommons.org/licenses/odbl/1.0/). The Open Database License (ODbL) is a license agreement intended to allow users to freely share, modify, and use this Dataset while maintaining this same freedom for others, provided that the original source and author(s) are credited.Alexandra Fernandes Costa, Salvatore Siciliano, Renata Emin-Lima, Bruna Maria Lima Martins, Maura Elisabeth Moraes Sousa, Tommaso Giarrizzo, José de Sousa e Silva Júnior

Supplementary material 3 S3 Movie Data type: MOV file Explanation note: Dolphin stranded at Praia da Travosa, Santo Amaro (MOV) This dataset is made available under the Open Database License (http://opendatacommons.org/licenses/odbl/1.0/). The Open Database License (ODbL) is a license agreement intended to allow users to freely share, modify, and use this Dataset while maintaining this same freedom for others, provided that the original source and author(s) are credited.north-eastern Brazilian coastsAlexandra Fernandes Costa, Salvatore Siciliano, Renata Emin-Lima, Bruna Maria Lima Martins, Maura Elisabeth Moraes Sousa, Tommaso Giarrizzo, José de Sousa e Silva Júnior
